# Plants promote mating and dispersal of the human pathogenic fungus *Cryptococcus*

**DOI:** 10.1371/journal.pone.0171695

**Published:** 2017-02-17

**Authors:** Deborah J. Springer, Rajinikanth Mohan, Joseph Heitman

**Affiliations:** 1 Department of Molecular Genetics and Microbiology, Duke University, Durham, North Carolina, United States of America; 2 Department of Biology, Hamilton College, Clinton, New York, United States of America; 3 Department of Medicine, Duke University Medical Center, Durham, North Carolina, United States of America; 4 Department of Pharmacology, Cancer Biology, Duke University Medical Center, Durham, North Carolina, United States of America; Research Institute for Children and the Louisiana State University Health Sciences Center, UNITED STATES

## Abstract

Infections due to *Cryptococcus* are a leading cause of fungal infections worldwide and are acquired as a result of environmental exposure to desiccated yeast or spores. The ability of *Cryptococcus* to grow, mate, and produce infectious propagules in association with plants is important for the maintenance of the genetic diversity and virulence factors important for infection of animals and humans. In the Western United States and Canada, *Cryptococcus* has been associated with conifers and tree species other than *Eucalyptus*; however, to date *Cryptococcus* has only been studied on live *Arabidopsis thaliana*, *Eucalyptus sp*., and *Terminalia catappa* (almond) seedlings. Previous research has demonstrated the ability of *Cryptococcus* to colonize live plants, leaves, and vasculature. We investigated the ability of *Cryptococcus* to grow on live seedlings of the angiosperms, *A*. *thaliana*, *Eucalyptus camaldulensis*, *Colophospermum mopane*, and the gymnosperms, *Pseudotsuga menziesii* (Douglas fir), and *Tsuga heterophylla* (Western hemlock). We observed a broad-range ability of *Cryptococcus* to colonize both traditional infection models as well as newly tested conifer species. Furthermore, *C*. *neoformans*, C. *deneoformans*, *C*. *gattii* (VGI), *C*. *deuterogattii* (VGII) and *C*. *bacillisporus* (VGIII) were able to colonize live plant leaves and needles but also undergo filamentation and mating on agar seeded with plant materials or in saprobic association with dead plant materials. The ability of *Cryptococcus* to grow and undergo filamentation and reproduction in saprobic association with both angiosperms and gymnosperms highlights an important role of plant debris in the sexual cycle and exposure to infectious propagules. This study highlights the broad importance of plants (and plant debris) as the ecological niche and reservoirs of infectious propagules of *Cryptococcus* in the environment.

## Introduction

Over 200 species of opportunistic fungi have been recognized as human pathogens over the last decade of which *Candida*, *Aspergillus*, and *Cryptococcus* species comprise the three most dominant opportunistic fungal infections worldwide [1]. Immunocompromised host status due to HIV/AIDS, diabetes, cancer, organ transplant, various prescribed medical treatments (immunosuppressive medications, steroids), and other genetic abnormalities are associated with increased risk of opportunistic fungal infections although infections in otherwise healthy individuals do occur less frequently [1, 2]. The development of new antifungals, highly active antiretroviral treatment (HAART), and combination therapy regimes have greatly increased long-term survival rates but infections due to opportunistic fungi remain difficult to control and frequently recur.

*Cryptococcus* is a dimorphic, environmental, basidiomycete that can occur as a yeast or in a filamentous/hyphal form that can cause fatal pulmonary and neurological infections if left untreated. Infections result from inhalation of infectious propagules (yeast or basidiospores) from the environment, which can have various length of incubation within the host prior to the development of overt disease. Progressive infections can disseminate, leading to systemic infections involving the brain and central nervous system that are often fatal, even with aggressive treatment. Historically, two major lineages, *Cryptococcus neoformans* and *Cryptococcus gattii*, of the pathogenic species complex were formerly described within the pathogenic species complex now delineated into seven species [3]. However, other species including *C*. *laurentii*, *C*. *albidus*, *C*. *uzbekistanensis*, *C*. *adeliensis*, *C*. *curvatus*, *C*. *magnus*, *C*. *humicolus*, *C*. *luteolus*, *C*. *macerans*, *C*. *flavescens*, and *C*. *uniguttulatus* have been associated with sporadic infections in immunocompromised individuals and other animals [4–7]. *Cryptococcus* is also associated with an ever increasing number of infections in seemingly healthy humans and animals in some regions of the world.

*C*. *neoformans* is now speciated into *C*. *deneoformans* (AFLP2, molecular type VNIV serotype D, and formerly *C*. *neoformans*), *C*. *neoformans* (molecular type VNI/AFLP1, VNII/AFLP1A/AFLP1B, and VNB/AFLP1; formerly *C*. *neoformans var*. *grubii*), and *C*. *deneoformans* and *C*. *neoformans* hybrids (VNIII/AFLP3). Five molecular types formerly recognized as *C*. *gattii* are currently described as five species, *C*. *gattii* (AFLP4, VGI, serotype B), *C*. *deuterogattii* (AFLP6, VGII, serotype B), *C*. *bacillisporus* (AFLP5, VGIII, serotype B and C), and *C*. *tetragattii* (AFLP7, VGIV, Serotype C) *C*. *decagattii* (AFLP10, VGIV). Rare interspecies hybrids have been identified between *C*. *deneoformans*, *C*. *neoformans*, *C*. *gattii*, and *C*. *deuterogattii* which are primarily isolated from clinical samples. Infections caused by *C*. *neoformans* are the most common worldwide and within the United States but in California *C*. *bacillisporus* VGIII is responsible for a majority of the naturally acquired cryptococcal infections in immunocompromised hosts, although VGI and VGII infections have also been reported [3, 8–11].

Infections associated with *Cryptococcosis* and *Aspergillus* are distinct from *Candida* because *Candida* is a natural component of the human microbiome, whereas *Aspergillus* and *Cryptococcus* are acquired from environmental exposure [12]. Infections initiated by *Cryptococcus* are unique from those initiated by *Aspergillus* because they require specific exposure to restricted ecological reservoirs, whereas spores of *Aspergillus* are ubiquitously dispersed in the air we breathe [13–15]. Periodic disease outbreaks of *Cryptococcus*, *Coccidioides immitis*/*posadasii*, *Blastomyces dermatitidis*, *Histoplasma capsulatum*, *Sporothrix schenckii*, or *Apophysomyces trapeziformis* are known to occur as a result of naturally occurring (earthquakes, tornadoes, or dust storms,) or human-initiated (construction, landscaping, or forestry practices) disturbances within specific ecological niches [16–26]. Therefore, the environmental reservoirs of *Cryptococcus*, the genetic diversity contained within these reservoirs, and the frequency of exposure to these reservoirs can greatly influence the risk of cryptococcal disease in immunocompromised individuals.

The environment plays an essential role as the exposure reservoir and breeding ground for the propagation and dispersal of infectious propagules (desiccated yeast and spores). Historically, *C*. *deneoformans/C*. *neoformans* was associated with pigeon and bird guano. Mating was first observed and described as the result of incubation on malt extract [27], sporulation agar [27], minimal medium minus thiamine agar [28], V8 agar [28], and more recently described on pigeon guano [29], in association with live plants [30], and on plant debris agars [31]. *Cryptococcus gattii* was distinguished from *C*. *neoformans* in the 1970’s and matings were observed and described on sporulation agar [32], V8 agar [33] and *A*. *thaliana* plants [30]. Laboratory matings of *C*. *gattii* (VGI, VGII, VGIII) are slower to progress and are less frequently observed than those involving *C*. *neoformans* isolates. Hybrid interspecies and intermolecular type matings and have been demonstrated in the laboratory but were associated with reduced viability [34, 35]. Less frequently, productive hybrid-matings can result in hybrid vigor, enhanced virulence, and increased antifungal resistance in hosts [34–36]. Opposite (MATα + MAT**a**) and same-sex (MATα + MATα) matings were first described for *C*. *neoformans/C*. *deneoformans* and later identified in *C*. *gattii* (VGI, VGII, VGIII) [27, 37]. The accumulation of 40 years of research now illustrates that both *C*. *neoformans*, *C*. *deneoformans*, *C*. *deuterogattii*, *C*. *bacillisporus*, and *C*. *gattii* can complete their lifecycle in the environment. Whereas *C*. *deneoformans* is more frequently isolated from bird guano, *C*. *gattii* (VGI, VGII, VGIII, and VGIV) is more frequently isolate from trees, and *C*. *neoformans* is associated with both bird guano and trees. Filamentation, mating, and the production of spores in association with plants may enhance nutrient accumulation and dispersal in the environmental niche [38]. Therefore, the genetic drivers of pathogenicity and virulence are a direct result of interactions and selective forces *Cryptococcus* encounters in the environment. In Western Canada and the Western United States, *Cryptococcus* has been associated with many tree species, but to date *Cryptococcus* has only been studied on live *A*. *thaliana*, *Eucalyptus sp*., and *Terminalia catappa* (Almond) seedlings. However *C*. *deuterogattii* (VGII) is frequently associated with conifers in the Western USA and information pertaining to the growth of *Cryptococcus* in these naturally associated plant species is lacking [30, 31, 39–42]. We therefore sought to extend the known interaction of *Cryptococcus* with plant host plants encountered within the United States, including live conifers and saprobic matter to further understand the role of plants in the infectious lifecycle and virulence of *Cryptococcus*.

## Methods

### *Arabidopsis thaliana* infection model

*Cryptococcus* cells were subcultured twice in yeast peptone dextrose (YPD) broth at 25°C shaking at 180 rpm. Cells were collected by centrifugation, washed in ddH_2_O, and resuspended to OD_600_ = 0.1 (~ 10^7^ cells/mL) in autoclaved ddH_2_O. Four whole leaves of three- to four-week old soil-grown *A*. *thaliana* plants (Col-0 wildtype, *jar1-1*, or *npr1-1*) were infiltrated with a suspension of *Cryptococcus* cells. Plants were incubated in 16 hours light at 24°C and 8 hours of dark at 20°C, or 12 hours of light and 12 hours of darkness.

One leaf per plant was harvested, homogenized in ddH_2_O, and plated onto YPD and Niger seed agar to analyze colony forming units (CFUs). Plates were incubated at 30°C for two to five days and CFUs were counted.

### Conifer, Mopane, and Eucalyptus infection models

Mopane (*Colophospermum mopane*, B and T World Seeds: http://b-and-t-world-seeds.com) and *Eucalyptus* seedlings (*Eucalyptus camaldulensis*, B and T World Seeds: http://b-and-t-world-seeds.com) were soil-grown from seed. Two- to four-year-old, non-sterile, soil-grown Douglas fir (*Pseudotsuga menziesii*) and Western hemlock (*Tsuga heterophylla*) seedlings were purchased from Musser Forest Inc. (https://www.musserforests.com). *Cryptococcus* cells were subcultured twice in YPD broth at 25°C with the appropriate drug selection nourseothricin [NAT 10 mg/L], neomycin [NEO 10 mg/L], or hygromycin [HYG 10 mg/L] respective to strain genotype (30°C, 180 rpm shaking). *Cryptococcus* cells were then collected by centrifugation, washed in ddH_2_O, and resuspended to OD_600_ = 0.1 (~ 10^7^ cells/mL) in autoclaved ddH_2_O.

Individual and mating mixtures of *C*. *deneoformans*, *C*. *neoformans* or *C*. *gattii*, *C*. *deuterogattii*, *or C*. *bacillisporus* were inoculated on three different branches or leaves of the same individual plant and replicated on three individual plants. For *Mopane*, *Eucalyptus*, Douglas fir, and Eastern hemlock experiments multiple leaves/branches of the same plant were used for different treatments, starting from top, ddH_2_O control, 3 branches for *Cryptococcus* inoculation (Strain 1, Strain 2, Strain 1 + Strain 2), and remaining branches served as inoculated controls. 20 μL of a OD_600_ = 0.1 *Cryptococcus* cell suspension (~2.0 x 10^5^ cells) were drop inoculated onto the adaxial surface of two to three Mopane or *Eucalyptus* leaves. Three individual branch tips of Douglas fir or Western hemlock seedlings (composed of multiple needles and emerging buds) were dipped into the OD_600_ = 0.1 cell suspension, excesses drips were blotted off with sterile Kimtech Science* KIMWIPES*, and allowed to air dry in a BSLIII hood. Additionally, one branch per tree was dip-inoculated with autoclaved ddH_2_O as experimental controls. Plants were incubated in an enclosed incubation chamber in 12 hours of light and 12 hours of dark that temperatures ranged from 30°C to 25°C respectively. Plant leaves or needles were harvested three weeks post-infection, homogenized in 1 mL autoclaved ddH_2_O and plated on YPD, YPD + NAT (100 mg/L), YPD + NEO (100 mg/L), and YPD + NAT (100 mg/L) + NEO (100 mg/L).

### Growth and mating on plant materials and agar

Freshly grown *Cryptococcus* cells were harvested from YPD agar plates and suspended in 1 mL autoclaved ddH_2_O and OD_600_ was measured. Cells were diluted to OD_600_ = 1.0. Then, 10 μL of each strain were spotted individually or in combination with the opposite mating partner on agar or directly on autoclaved plant materials placed on top of 2% water agar (20 g Difco Bacto^®^ agar/L) in petri-plates. Plant-based agars contained 30 g Difco Bacto^®^ agar + 20 g ground plant materials (fresh *A*. *thaliana* plants, Black cherry (*Prunus serotina*) wood chips, Almond, Coco, Long leaf pine (*Pinus palustris*) needles, pine wood chips, Sugar maple (*Acer saccharum*) wood chips, Western red cedar (*Thuja plicata*), Douglas fir (*Pseudotsuga menziesii*), Western hemlock (*Tsuga heterophylla*) wood chips, or Mopane wood chips + 1 mL 20% glucose per L and pH was not adjusted. Plant materials were ground uniformly with a coffee bean grinder that was cleaned thoroughly with water and ethanol between each preparation. Black cherry and sugar maple wood chips (obtained from Dr. Paul Manion, Cazenovia, New York); *A*. *thaliana* plants (Dong lab, Duke University); Almonds (Whole, dry, and non-salted, Kroger brand); pine shavings (Petco, bedding); Douglas fir, Western red cedar, and Hemlock chips (obtained from Jamie, Murry, GEM Shavings, Auburn, Washington) were homogenized in a spice grinder. Coca agar was composed of 20 g Hershey’s powder cocoa + 20 g Difco Bacto^®^ agar and pH was not adjusted. V8 agar pH 5, V8 agar pH 7, MS agar, Niger seed agar, and Filament agar were made following standard methodology. V8 fusion agar was made substituting V8 Strawberry Banana fusion juice (50 mL) for standard V8 juice + 0.5 g KH_2_PO_4_ + 30 g Difco Bacto^®^ agar, and pH was adjusted to pH 5 or pH 7. Fusion agar consisted of 100 mL V8 Strawberry Banana fusion juice + 30 g Difco Bacto^®^ agar and pH was not adjusted.

Tree needles (Hemlock, Long leaf pine, Eastern red cedar), leaves (Sugar maple), wood chips (Black cherry), Pine button plug (General Unfinished Flat Head no.315038 pine, Home Depot), or Oak button plugs (General Unfinished Flat Head no.313038 oak, Home Depot) were autoclaved, allowed to cool overnight, and aseptically placed in petri plates on top of 2% water agar before being inoculated with 15 μL of H99α, KN99**a**, or H99α + KN99**a** mating mixture; JEC21α, JEC20**a**, or JEC21α + JEC20**a** mating mixture; or NIH444α, NIH194**a**, or NIH444α + NIH194**a** mating mixture. Plates were incubated in a dark drawer at room temperature and observed weekly for the production of hyphae or basidia.

### *In vivo* murine model

Six-week-old female A/JCr mice (Cat. No. 01A24, NCI-Frederick) or male BALB/c mice (Cat. No. 01B05, NCI-Frederick) were used. All animal studies were conducted in the Division of Laboratory Animal Resources (DLAR) facilities at Duke University Medical Center (DUMC) and animals were handled according to the guidelines defined by the United States Animal Welfare Act and in full compliance with the DUMC Institutional Animal Care Use Committee (IACUC). Animal models were reviewed and approved by DUMC IACUC under IACUC protocol # A217-11-08. Mice were acclimated in the facility for one week prior to infection and were housed in cages at 21°C and 50% humidity with a 12 hrs. light/12 hrs. dark cycle and given ample food and water daily. *Cryptococcus* cells were grown on *Arabidopsis* agar (20 g/L *A*. *thaliana* plants + 20 g/L agar) at 30°C for six days. Cells were harvested by scraping and washing from the agarose plates with autoclaved ddH_2_O and resuspended in 10 mL of autoclaved ddH_2_O. Cell were counted with a hemocytometer and resuspended to 2 x 10^6^ cells/mL. All mice were sedated with Nembutal (sodium pentobarbital) prior to intranasal inoculation with 10^6^ Cryptococcus cells in 40 μl. We expect the mice will become ill from inoculation with *Cryptococcus* which may exhibit as social isolation, lack of grooming, weight loss, loss of balance, inability to feed, loss of sternal recumbency, limb paralysis, seizures, convulsion, and coma. Animals were monitored daily by the primary author and Duke University DLAR staff for signs of disease development, distress, or suffering as described previously and were euthanized utilizing CO_2_ when weight loss ≥ 15% of original body weight or they exhibited neurological symptoms or cranial swelling. Kaplan-Meier survival curves were constructed by GraphPad Prism version 6.03 (Windows, GraphPad Software, La Jolla California USA, www.graphpad.com).

### Electron microscopy

To examine mating reactions for morphological features associated with mating, scanning electron microscopy studies were conducted. First, one centimeter square blocks of agar, whole needles, or sectioned leaf tissues were collected and fixed in 2% glutaraldehyde (Electron Microscopy Sciences, EMS, Hatfield, PA, USA) with 0.05% malachite green oxalate (EMS) in 0.1 M sodium cacodylate buffer and incubated at 4°C until further processing. The fixation buffer was removed, and blocks were dehydrated by ethanol series, critical point dried (Pelco CPD2, Ted Pella, Inc., Redding, California, USA), sputter coated, and imaged with the FEI XL30 SEM-FEG (FEI Company, Hillsboro, Oregon, USA) at the electron microscopy facility at North Carolina State University.

## Results

### *Cryptococcus* colonizes live *Arabidopsis thaliana* plants

**Fig 1:**
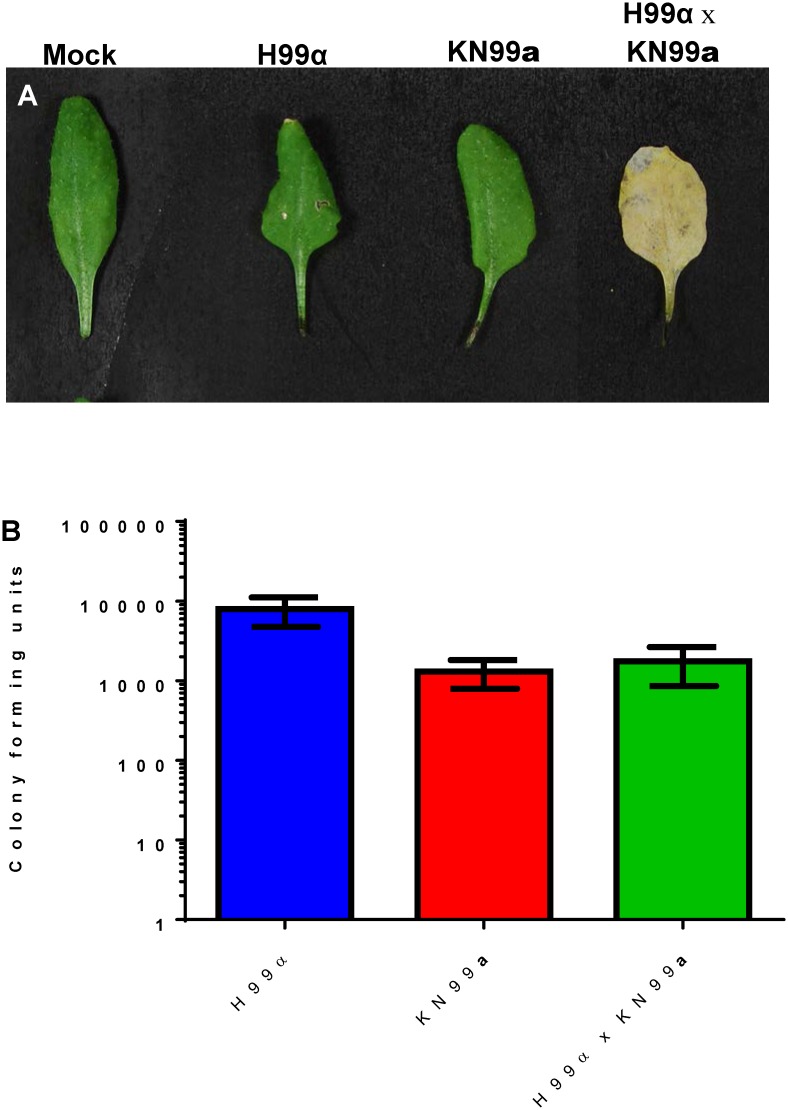
*Cryptococcus neoformans* (VNIV) can colonize mature soil grown *Arabidopsis thaliana*. (A) Mating mixtures can induce chlorosis, (B) Colony forming units (CFUs + SEM) indicate that both individual and mating strains of *Cryptococcus* can colonize *A*. *thaliana* plants. Chlorosis was only associated with mated mixtures of *C*. *neoformans* (VNI).

**Fig 2:**
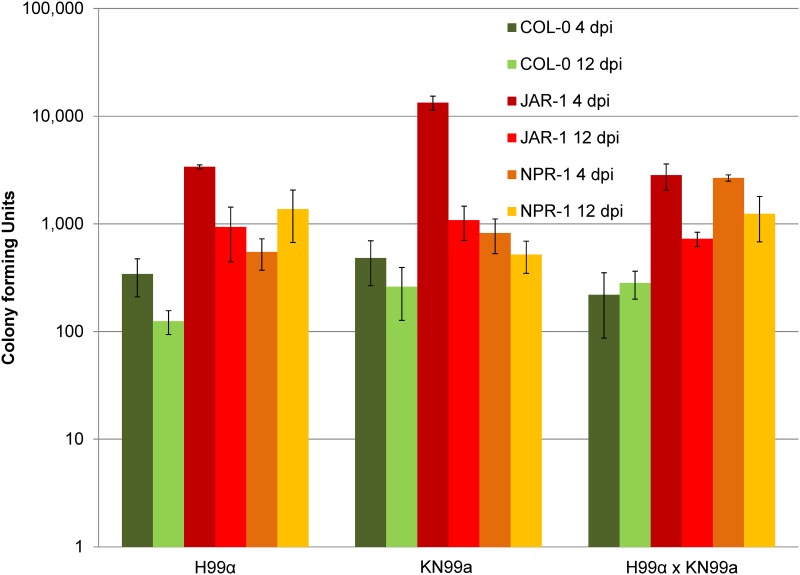
*Arabidopsis thaliana* mutants display increased susceptibility to *Cryptococcus* colonization. *A*. *thaliana jar1-1* mutants display increased colonization by *C*. *neoformans* (VNI) at four and twelve days post infection (dpi). *A*. *thaliana npr1-1* mutants display increased colonization to mixed *C*. *neoformans* infection at 4 and 12 days post-infection. Error bars represent CFUs + SEM.

*Cryptococcus* can colonize a broad variety of live plants. We demonstrate that *C*. *neoformans* (VNIV, Fig 1) *C*. *deuterogattii* (VGII), and *C*. *gattii* (VGI, S1 Fig) can colonize mature soil grown *A*. *thaliana* plants as previously described [30, 39]. Mature soil grown *A*. *thaliana* plants were colonized more readily by H99α in comparison to KN99**a** (p < 0.0001). Survival and colonization of *A*. *thaliana* by *Cryptococcus* is strain-dependent (S1 Fig). Colonization of *A*. *thaliana* by *C*. *neoformans* is not dependent on laccase, capsule, or calcineurin (p > 0.100). However, we observed reduced colonization of *C*. *deuterogattii* (VGII) calcineurin (*cna1*Δ) mutant strains on *A*. *thaliana* plants (p < 0.001) S1 Fig). Chlorotic symptoms were only observed in leaves inoculated with mated pairs (MAT**a** x MATα) and were not correlated with increased fungal colonization (Fig 1, KN99**a** versus H99α x KN99**a**, p > 0.100). *A*. *thaliana* mutants *jar1-1* (p < 0.001) and *npr1-1* (12 dpi and H99α x KN99**a** p < 0.001) were more permissible to *C*. *neoformans* colonization in comparison to Wild-type (Col-0) (Fig 2). Scanning electron microscopy indicates that *C*. *neoformans* colonizes live soil-grown wild type and mutant lines of *A*. *thaliana* (S2 Fig). Wild-type (Col-0) and *jar1-1 A*. *thaliana* seedlings inoculated with mated mixtures of *C*. *neoformans* (H99α x KN99**a**) displayed reduced growth and increased pigmentation (likely anthocyanin) in comparison to seedlings inoculated with individual strains (S3 Fig).

### *Cryptococcus* colonizes live Douglas fir, Western hemlock, Mopane, and *Eucalyptus* seedlings

**Fig 3:**
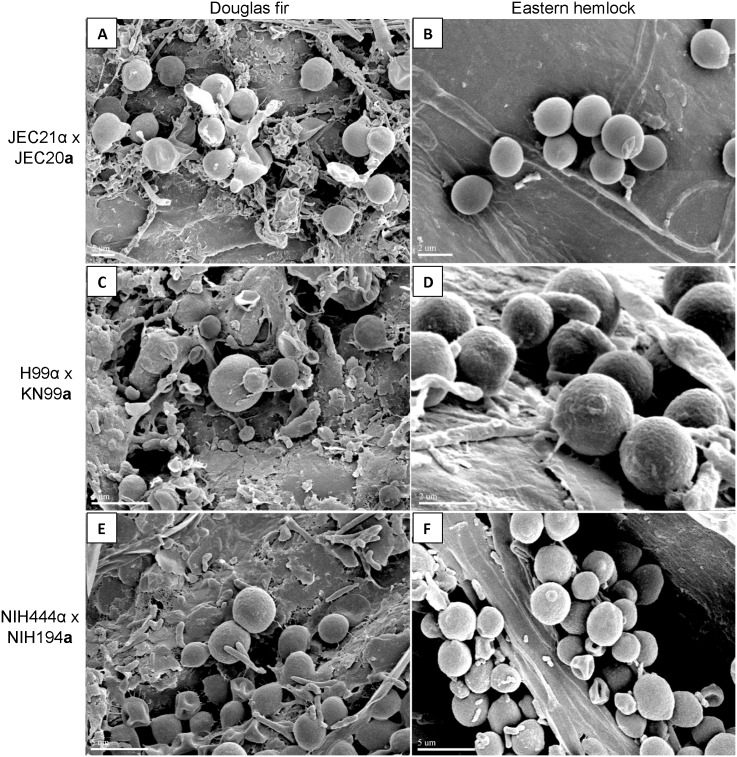
*Cryptococcus* can colonize live Douglas fir and Western hemlock trees. Scanning electron micrographs are shown of mixed mating strains of (A) *Cryptococcus deneoformans* (VNIV) producing filaments on Douglas fir trees, and (B) colonizing Eastern hemlock trees. Colonization of *Cryptococcus neoformans* (VNIV) on Douglas fir (C) or Western hemlock (D), and of *C*. *deuterogattii* (VGII) *x C*. *gattii* (VGI) on Douglas fir (E) or Hemlock (F) are shown. Scale bar = 5 μm.

**Fig 4:**
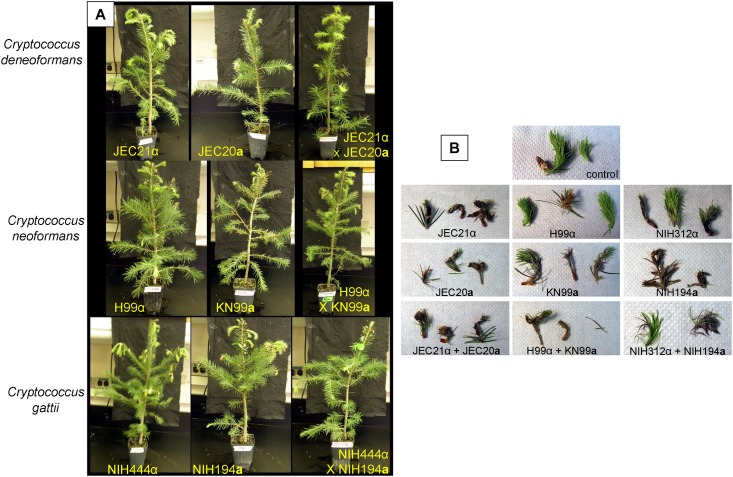
Douglas fir and Eastern hemlock infection model at one week post infection. (A) Top row displays Douglas fir inoculated with (A) *C*. *deneoformans* JEC21α, JEC20**a**, and JEC21α + JEC20**a** mixed; Middle row shows, *C*. *neoformans* H99α, KN99**a**, and H99α + KN99**a** mixture; Bottom row shows, C. *deuterogattii* (NIH312α), *C*. *gattii* (NIH194**a)**, and NIH312α + NIH194**a** mixture. (B) Inoculated buds and needles predominantly appear green and healthy but some browning or loss of needles are observed for both individually inoculated strains and mated mixtures. (B) Left column shows, *C*. *deneoformans* JEC21α, JEC20**a**, and JEC21α + JEC20**a** mixed; Middle column under labeled control shows, *C*. *neoformans* H99α, KN99**a**, and H99α + KN99**a** mixture; and right column shows, *C*. *deuterogattii* (NIH312α), *C*. *gattii* (NIH194**a)**, and NIH312α + NIH194**a** mixture.

**Fig 5:**
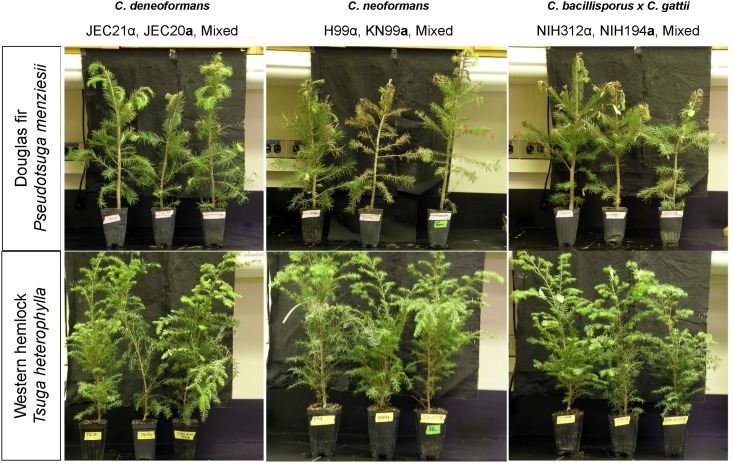
*Cryptococcus* can infect conifers and shows progressive disease associated symptoms. Conifer infection model at four weeks post-infection displays exacerbated needle browning, bud drooping, and needle dropping consistent with infection. Douglas fir (top row) and Eastern hemlock (bottom row) four weeks post inoculation with, (left column) *C*. *deneoformans* JEC21α, JEC20**a**, and JEC21α + JEC20**a** mixed; (middle column) *C*. *neoformans* H99α, KN99**a**, and H99α + KN99**a** mixture; (right column) *C*. *bacillisporus* (NIH312α), *C*. *gattii* (NIH194**a)**, and NIH312α + NIH194**a** mixture. Douglas fir displays more plant infection symptoms in contrast to Eastern hemlock.

*C*. *deneoformans*, *C*. *neoformans*, *C*. *deuterogattii*, *C*. *bacillisporus*, and *C*. *gattii* can colonize Douglas fir and Western hemlock seedlings. Electron microscopy demonstrates that *C*. *deneoformans* (VNIV), *C*. *neoformans* (VNI), *C*. *deuterogattii* (VGII), and *C*. *gattii* (VGI) can colonize live Douglas fir and Western hemlock needles in mixed communities with other naturally acquired microorganisms (Fig 3). Douglas fir seedlings appeared healthy at one week post inoculation (Fig 4), but developed browning needles and progressive disease symptoms on young buds by three weeks (Fig 5). Disease symptoms progressively developed with prolonged incubation. Obvious disease symptoms were not observed on Western hemlock seedlings even after prolonged incubation (Fig 5). Douglas fir seedlings inoculated with *C*. *neoformans*, *C*. *bacillisporus* (VGIII), and *C*. *gattii* (VGI) displayed more severe needle browning and bud symptoms than those inoculated with *C*. *deneoformans*. Disease symptoms were associated with both mated mixtures and individually inoculated strains. Disease symptoms were not observed on *Eucalyptus* or Mopane seedlings inoculated with *C*. *deneoformans*, *C*. *neoformans*, *C*. *bacillisporus*, or *C*. *gattii* (S4 Fig).

**Fig 6:**
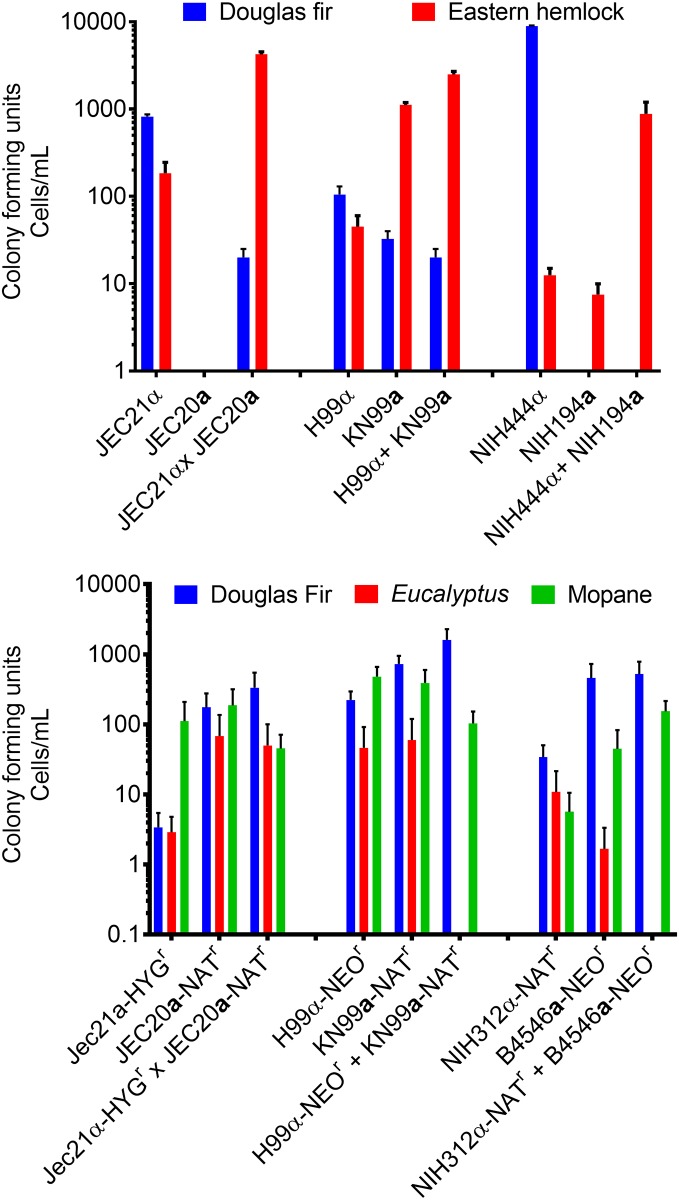
Conifer trees are susceptible to colonization by *Cryptococcus*. Colony forming units (CFUs + SEM) at three weeks post-inoculation are shown. (A) *Cryptococcus* cells were recovered from Douglas fir and Eastern hemlock seedlings. *C*. *neoformans* was recovered from both Douglas fir and Eastern hemlock; however, recovery of *C*. *deneoformans*, *C*. *gattii*, *C*. *bacillisporus*, *C*. *deuterogattii* strains was inconsistent. In a plant infection trial, Douglas fir, *Eucalyptus*, and Mopane infection models were compared utilizing engineered strains containing various drug-resistant cassettes. *Cryptococcus* cells were recovered from *Eucalyptus*, Mopane, and Douglas fir infected with individual and mated strains. Error bars represent +SEM.

**Fig 7:**
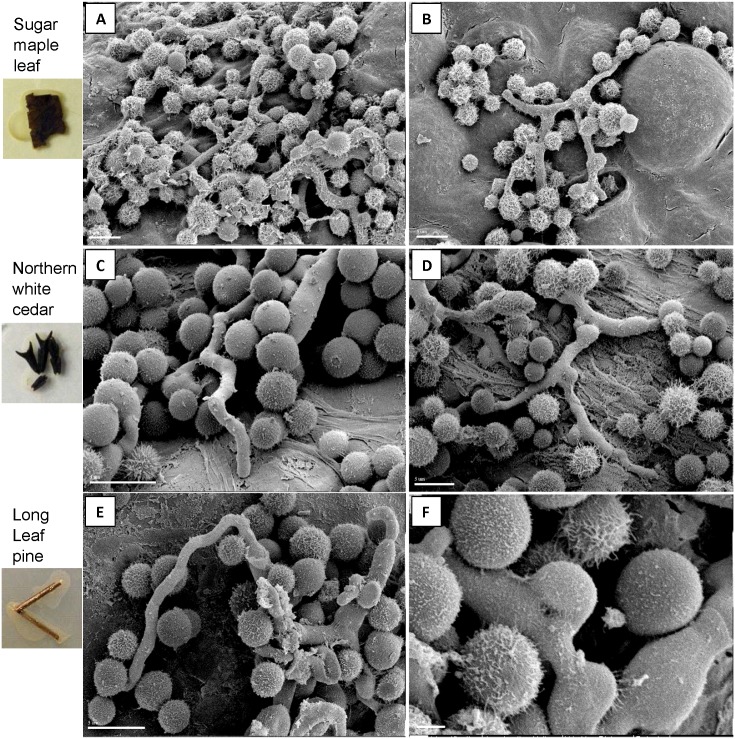
*Cryptococcus* can grow saprobically and mate on dead plant materials. Scanning electron micrographs depict *C*. *neoformans* (VNI) displaying robust filamentation on sugar maple leaves (top row), northern white cedar needles (middle row) and long leaf pine needles (bottom row). Fused clamp connections are indicative of productive matings (arrow).

*Cryptococcus* recovery from infected tree seedlings appears to be plant- and strain-dependent, but *C*. *deneoformans*, *C*. *neoformans*, *C*. *deuterogattii C*. *bacillisporus*, and *C*. *gattii* were able to colonize tree seedlings (Fig 6). *C*. *neoformans* were more consistently recovered from inoculated Douglas fir and Western hemlock seedlings compared with *C*. *deneoformans* and *C*. *deuterogattii C*. *bacillisporus*, or *C*. *gattii* (Fig 6a). *Cryptococcus* cell recovery from *Eucalyptus* was inconsistent between strains (Fig 6b). *Cryptococcus* recovery was enhanced by utilizing *Cryptococcus* strains containing a drug-resistance marker (Fig 6b). No double drug resistant colonies were isolated from trees inoculated with mixed mating strains. Furthermore, we observed by light microscopy (S5 Fig) and scanning electron microscopy (Fig 7) that *Cryptococcus* can grow saprobically, filament (Oak, Hemlock, Maple, and Long leaf pine), and mate on dead plant materials including Sugar maple leaves and needles of Cedar and Long leaf pine (Fig 7).

### *Cryptococcus* can mate on agar containing plant materials

**Fig 8:**
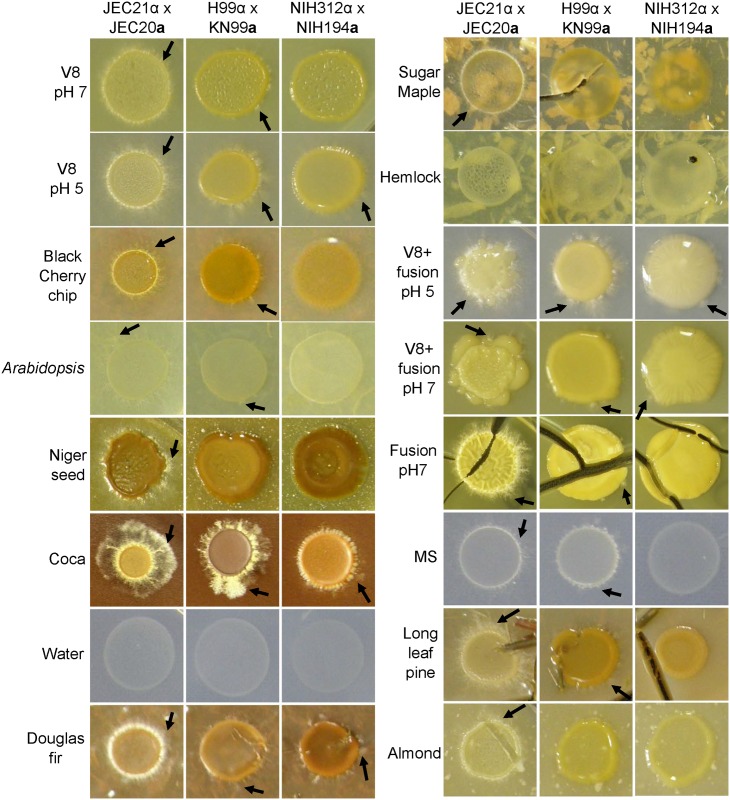
Enhanced mating of *Cryptococcus* is observed on plant material-based agars. *C*. *deneoformans* and *C*. *neoformans* display filamentation (arrows) around the periphery of the colony on many types of plant-based agars at four weeks post inoculation. Mating and the production of basidia were confirmed (S7, S8, S9 and S10 Figs). Mating of *C*. *bacillisporus* x *C*. *gattii* was less prolific and was confirmed based on microscopic observation of hyphae, basidia, and spores. (S7, S8, S9 and S10 Figs). For *C*. *bacillisporus* x *C*. *gattii* arrows highlight detectable areas of filamentation with confirmation by light microscopy.

*C*. *neoformans* is highly fertile and prolifically mates on plant agars. Mating of *C*. *deneoformans* and *C*. *neoformans* was observed on Black cherry chip, *Arabidopsis*, Niger seed, Coca, Douglas fir, Sugar maple, Longleaf pine, and Almond agars, as well as on classic V8 and MS agar and newly concocted V8-fusion and Fusion agars (Fig 8 and S6 Fig). Strains of *C*. *bacillisporus* (VGIII) *x C*. *gattii (VGI)* had fewer observed matings and were slower to mate on many of the tested media (Fig 8 and S6 Fig). Mating of *C*. *bacillisporus* (VGIII) *x C*. *gattii (VGI)* was observed on Douglas fir, Cocoa, V8-fusion, Fusion, and classic V8 (pH 5) agar (Fig 8). Filamentation and basidia observed by direct microscopic analysis confirmed the mating of *C*. *deneoformans*, *C*. *neoformans*, and *C*. *bacillisporus* (VGIII) *x C*. *gattii (VGI)*. S7 Fig depicts colony morphology and S8 Fig displays photomicrographs of the production of hyphae and basidia on standard mating media in contrast to mating on newly described plant-based agars displayed in S8 Fig (colony morphology) and S10 Fig (filamentation and basidia).

### *Cryptococcus gattii* can grow in extract broth

*C*. *deuterogattii* and *C*. *bacillisporus*, can grow and proliferate in *A*. *thaliana* and Pigeon guano extract broth. The OD of *C*. *deuterogattii* and *C*. *bacillisporus* cells followed normal growth dynamics over 72 hours, logarithmically increasing over 24 hours before reaching stationary phase (S11a Fig). Viable cells were obtained at the conclusion of the growth curve (S11b Fig). *A*. *thaliana* and pigeon guano extract broth have no additional nutrients, and growth was similar to YPD broth over 8 hours but then rapidly reached stationary phase around 12 hours; viable cells were recovered at the termination of the growth curve at 72 hours.

### The impact on virulence due to growth on *Arabidopsis* agar is strain dependent

**Fig 9:**
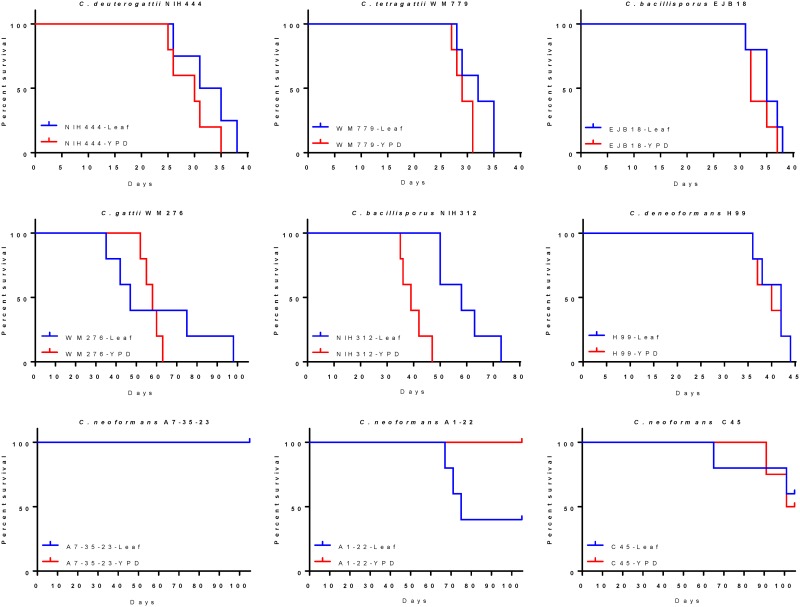
Strain dependent changes in the virulence of *Cryptococcus* as a result of passage on plant-based agars are depicted. Kaplan-Meier survival curves of nine *Cryptococcus* strains grown on *Arabidopsis* agar are shown. *C*. *bacillisporus* NIH312 (E) demonstrated reduced virulence and *C*. *neoformans* A1-22 (H) displayed increased virulence following one week of growth on *Arabidopsis* plant agar. No significant differences were observed between YPD agar and *Arabidopsis* agar for any other strain.

*C*. *deneoformans*, *C*. *neoformans* and *C*. *gattii*, *C*. *deuterogattii*, *C*. *bacillisporus*, and *C*. *tetragattii* strains were grown on *Arabidopsis* agar for one week, harvested from plates, and tested for virulence in the intranasal murine model (Fig 9). Hypervirulence as a result of growth on plant materials was only observed for *Cryptococcus neoformans* isolate A1-22 (Fig 9). Hypovirulence as a result of growth on *Arabidopsis* agar was observed for *C*. *bacillisporus* VGIII isolate NIH312 (Fig 9). No other statistically significant differences (P > 0.05) were observed for growth on YPD agar or *Arabidopsis* agar were observed for NIH444, WM779, EJB18, WM276, H99, A7-35-33 or C45 (Fig 9).

## Discussion

The human pathogen, *Cryptococcus*, can utilize plant hosts as reservoir for mating and dispersal. It is presently understood that *C*. *deneoformans*, *C*. *neoformans*, and VGI, VGII, and VGIII crosses can colonize and mate on *A*. *thaliana* in the laboratory setting, but little is known regarding the ability of *Cryptococcus* to colonize trees that it is known to associate with in nature other than limited reports of infection of almond and *Eucalyptus* seedlings. In this study, we demonstrated that *Cryptococcus* can colonize live and dead plant tissues from a number of natural plant hosts and that these plant materials can trigger the transition of yeast cells to a filamentous stage supporting the important role of these hosts in the environmental lifecycle of *Cryptococcus*. Prolific filamentation of *Cryptococcus* was observed on dead sterilized plant materials and on water agar containing plant matter suggesting that *Cryptococcus deneoformans*, *C*. *neoformans*, *C*. *gattii*, *C*. *deuterogattii*, *C*. *bacillisporus*, and *C*. *tetragattii* may interact with plants at various stages of their lifecycle including seedlings, mature plants, to necrotic and dead plant matter by adapting different life strategies as a biotroph, saprobe, or as pathogen. The ability of *Cryptococcus* to transition to alternative life strategies in association with host may be impacted by the presence of both mating types because plant pathogenicity and the filamentation, mating, and the generation of double drug resistant progeny by *Cryptococcus* was only observed under those circumstances [30]. However, we observed filamentation, production of clamp connections, and basidia by *Cryptococcus* in saprobic association with sterile plant matter and agar supplemented with plant matter indicating the sexual lifecycle can also be completed in saprobic association with plant matter. It is possible that the pathogenic *Cryptococcus species* (VN and VG inclusive) could form epiphytic and endophytic associations with plants because *C*. *laurentii* and *C*. *albidus*, *C*. *flavus*, *C*. *podzolicus*, *C*. *hungaricus*, and other *Cryptococcus sp*. have been reported to form non-pathogenic associations with a variety of host plants [43–46].

Consistent with previous reports we observed *A*. *thaliana* is readily colonized by *C*. *deneoformans*, *C*. *neoformans*, *C*. *gattii*, *C*. *deuterogattii*, and *C*. *bacillisporus*. Evidence has been previously reported that *Cryptococcus* mating triggers a jasmonic acid (JA)-dependent defense response in Arabidopsis and that Salicylic acid (SA) signaling pathway involving NPR1 is suppressed [30]. Here, we show that both JA and SA signaling pathways reduce the colonization of *Cryptococcus* in *Arabidopsis* as the JA and SA signaling mutants, *jar1-1* and *npr1-1*, both show increased fungal colonization. This is consistent with previous reports that *Arabidopsis* SA signaling or synthesis mutants *eds1* (enhanced disease susceptibility 1; lipase/signal transducer/triacylglycerol lipase), *nahG* (transgenic line degrading salicylic acid; SA), *sid2* (SA-induction deficient), and *npr1* (nonexpressor of PR genes 1; pathogenesis-related 1) are all more susceptible to *Cryptococcus* infection [39, 41]. Other mutant *A*. *thaliana* ecotypes *rpm1* (resistance to *Pseudomonas syringae pv maculicola* 1), *pad4* (phytoalexin deficient 4), and *Atprn1* (PRN1; one of four members of an iron-containing subgroup of the cupin superfamily, accumulates flavonoids) that are components of SA-mediated pathways, were also observed to be more susceptible to *Cryptococcus* infection [39, 41]. The data suggest that both the SA and JA pathways are required to hinder the colonization and growth of *Cryptococcus* in *Arabidopsis* and are not necessarily antagonistic to each other consistent with resistance to the pathogenic fungus *Fusarium graminearum* [47].

Furthermore, in laboratory trials, *Cryptococcus* mutants (*ste12*α, *cap59*, *lac1*) show reduced ability to colonize *A*. *thaliana* plants and could be differentially regulated between species and molecular types as in mating and virulence [41, 48]. Laccase was not essential for the colonization of *A*. *thaliana* by *C*. *deuterogattii* [42] but was important for virulence in *C*. *neoformans*, suggesting that different *Cryptococcus* species may have adapted different strategies to cope with the evolutionary pressures encountered in the plant ecological niche [41].

The interactions between *Cryptococcus* and plants are likely dependent on both the environmental conditions, the genetics of *Cryptococcus*, and the genetics of the host, *A*. *thaliana*. Conserved factors that are utilized for both plant colonization and virulence within animal host could be maintained in the natural ecological niche providing for the natural reservoirs of infectious genotypes. Furthermore, both yeast and mated co-cultures were observed to colonize live plants and plant tissues that were associated with the appearance of disease symptoms [30, 39, 42].

In this study, we demonstrate the ability of *C*. *deneoformans*, *C*. *neoformans* and *C*. *gattii*, *C*. *bacillisporus*, *C*. *deuterogattii*, and *C*. *tetragattii* to colonize live non-sterile Douglas fir and Hemlock trees and to undergo saprobic filamentation, mating, and the production of spores on dead plant material, implicating the potential for long-term association of *Cryptococcus* with plants. Therefore, plants may serve an important role in maintaining, optimizing, and enhancing virulence factors during the environmental lifecycle of the opportunistically acquired human pathogen, *Cryptococcus*.

## Supporting information

S1 Fig *C*. *deneoformans*, *C*. *neoformans*, *C*. *gattii*, *C*. *deuterogattii*, and *C*. *bacillisporus* readily colonize mature soil grown *A*. *thaliana* plants. *LAC1* and *CNA1* are not required for *A*. *thaliana* colonization.
(DOCX)

S2 Fig *Cryptococcus neoformans* (VNI) can colonize *A*. *thaliana*. Scanning electron microscopy indicates active colonization of mutant (*jar1-1*, *npr1-1*) and wildtype (Col-0) *A*. *thaliana* plants.
(DOCX)

S3 Fig Immature, agar grown, *A*. *thaliana* seedlings are more sensitive to cryptococcal infections. (A) Mating mixtures can alter growth of immature *A*. *thaliana* seedlings. (B) *jar1-1* seedlings display increased deleterious symptoms associated with *Cryptococcu*s *neoformans* (VNI) colonization.
(DOCX)

S4 Fig Leaves of *Eucalyptus* and Mopane do not show deleterious symptoms associated with infection of *C*. *neoformans*, *C*. *deneoformans*, or *C*. *bacillisporus*. Leaves from two individual *Eucalyptus* or Mopane seedlings were harvested at three weeks post inoculation.
(TIF)

S5 Fig *Cryptococcus* can filament and mate in saprobic association with plant materials. Robust filamentation of *C*. *deneoformans* is observed in association with black cherry chips and oak but limited filamentation is also observed in association with Sugar maple leaf, Hemlock needle, and long leaf pine needle. Robust filamentation of *C*. *neoformans* was observed in association with oak and limited filamentation with Sugar maple and Long leaf pine.
(DOCX)

S6 Fig *C*. *deneoformans* and *C*. *neoformans* sexual reproduction on plant material based agars is shown. Mating of *C*. neoformans is observed in as little as five to seven days post-inoculation. Detectable mating of *C*. *bacillisporus* (VGIII) x *C*. *gattii* (VGI) took longer, no filamentation or mating was observed one week post-inoculation. Red boxes denote hyphal growth and mating by light microscopic observation of basidia.
(DOCX)

S7 Fig Colony morphology and filamentation were observed along colony edges on standard laboratory mating media. *C*. *deneoformans* and *C*. *neoformans* produces filaments more prolifically than *C*. *bacillisporus* (VGIII) x *C*. *gattii* (VGI) on most media. Newly created fusion media and mixed V8-fusion media induce robust filamentation and mating of *C*. *bacillisporus* (VGIII) x *C*. *gattii* (VGI).
(DOCX)

S8 Fig Basidia are associated with filamentation on novel and standard mating media. *C*. *deuterogattii* (VGII) x *C*. *gattii* (VGI), *C*. *deneoformans*, and *C*. *neoformans* robustly form basidia and spores on newly synthesized fusion and V8-fusion blend media.
(TIF)

S9 Fig *Cryptococcus deneoformans* and *C*. *neoformans* colonies on plant based agars display robust filamentation phenotypes similar to those observed on standard mating media. *C*. *bacillisporus* (VGIII) x *C*. *gattii* (VGI) only sparsely filamented on A*rabidopsis*, black cherry, Coca, Long leaf pine, Sugar maple, and hemlock agars (S7 Fig).
(DOCX)

S10 Fig *Cryptococcus deneoformans* and *C*. *neoformans* filaments and forms basidia as a result of mating on plant based agars. *C*. *bacillisporus* (VGIII) x *C*. *gattii* (VGI) also produced basidia on *Arabidopsis*, black cherry, Coca, Sugar maple, and hemlock agars.
(DOCX)

S11 Fig Growth dynamics of *C*. *bacillisporus* and *C*. *deuterogattii* in broth are shown. (A) Reduced growth in *Arabidopsis* or Pigeon guano extract broth. (B) Colony forming units vs absorbance at OD_600_ confirms a higher level of viable cells in YPD broth vs *Arabidopsis* of pigeon guano extract broth.
(DOCX)
