# Supplementary figures and images for: Plants promote mating and dispersal of the human pathogenic fungus Cryptococcus
Source: PLoS One. 2017 Feb 17;12(2):e0171695. doi: 10.1371/journal.pone.0171695 (PMC5315327; doi:10.1371/journal.pone.0171695)

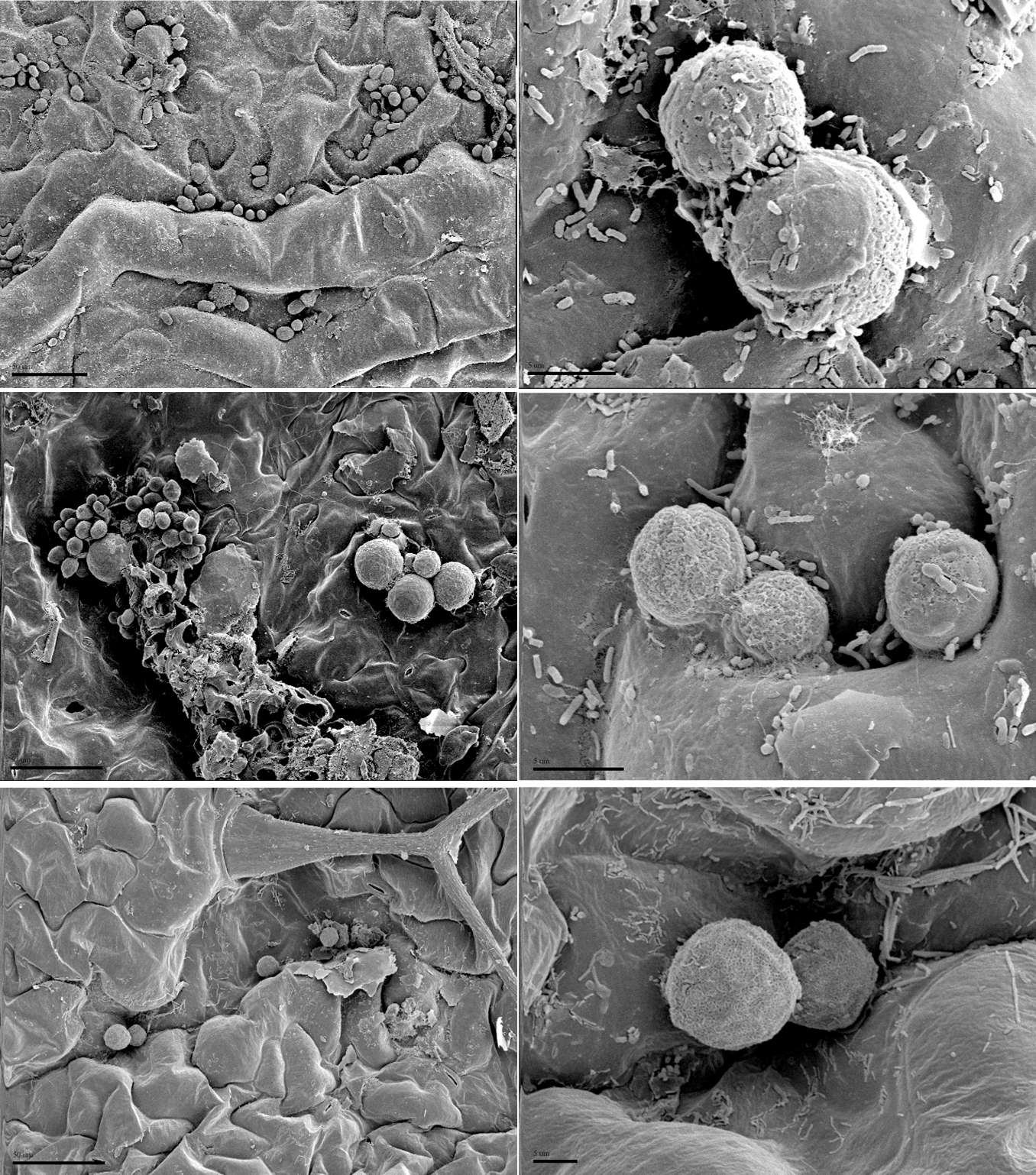


**Supplemental Figure 2**

*Arabidopsis*

NPR-1

*Arabidopsis*

JAR-1

*Arabidopsis*

COL-0

Supplement: S2 Fig — Scanning electron microscopy indicates active colonization of mutant (jar1-1, npr1-1) and wildtype (Col-0) A. thaliana plants. (DOCX) [file pone.0171695.s002.docx]

**JAR-1**

**COL-0**

**H99α + KN99a**

**KN99a**

**H99α**

**Mock**


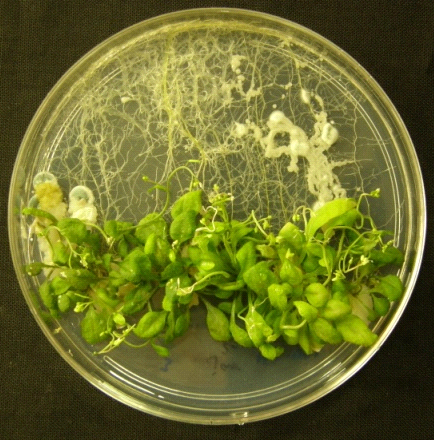

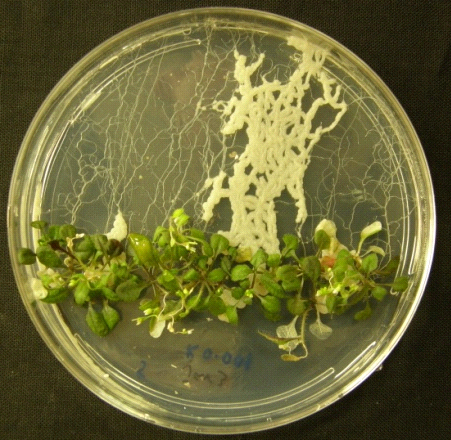

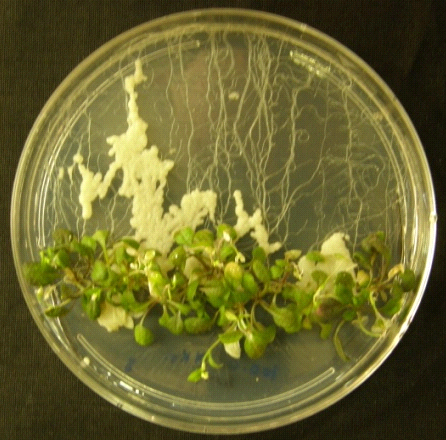

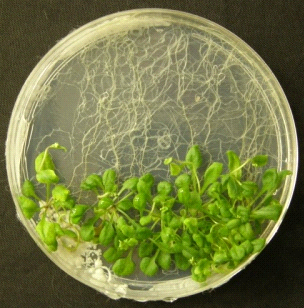

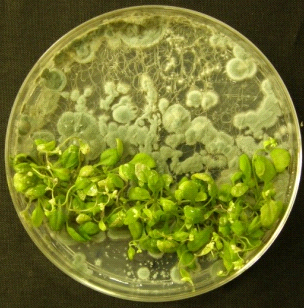

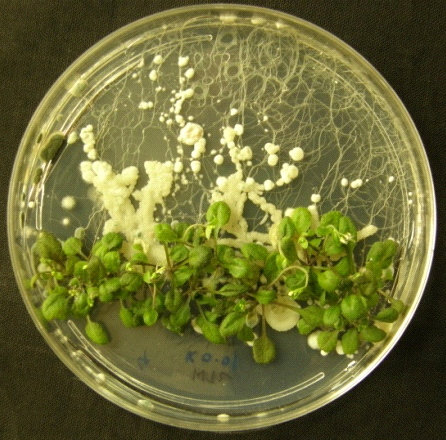

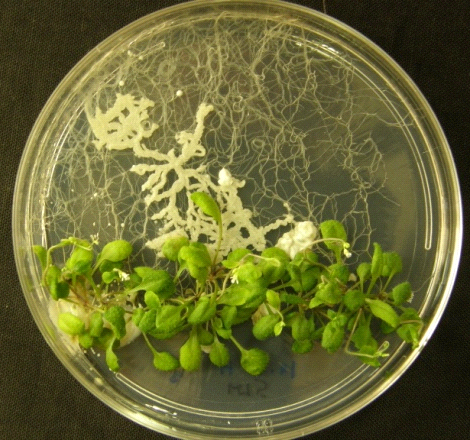

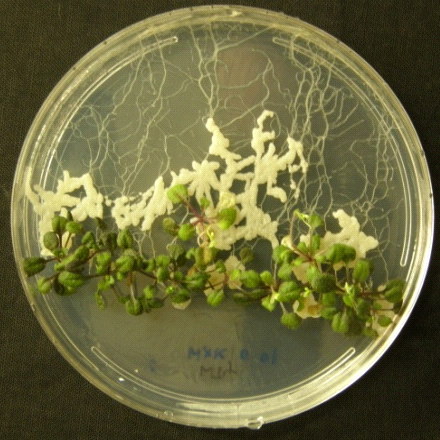


**Supplemental Figure 3**

Supplement: S3 Fig — (A) Mating mixtures can alter growth of immature A. thaliana seedlings. (B) jar1-1 seedlings display increased deleterious symptoms associated with Cryptococcus neoformans (VNI) colonization. (DOCX) [file pone.0171695.s003.docx]

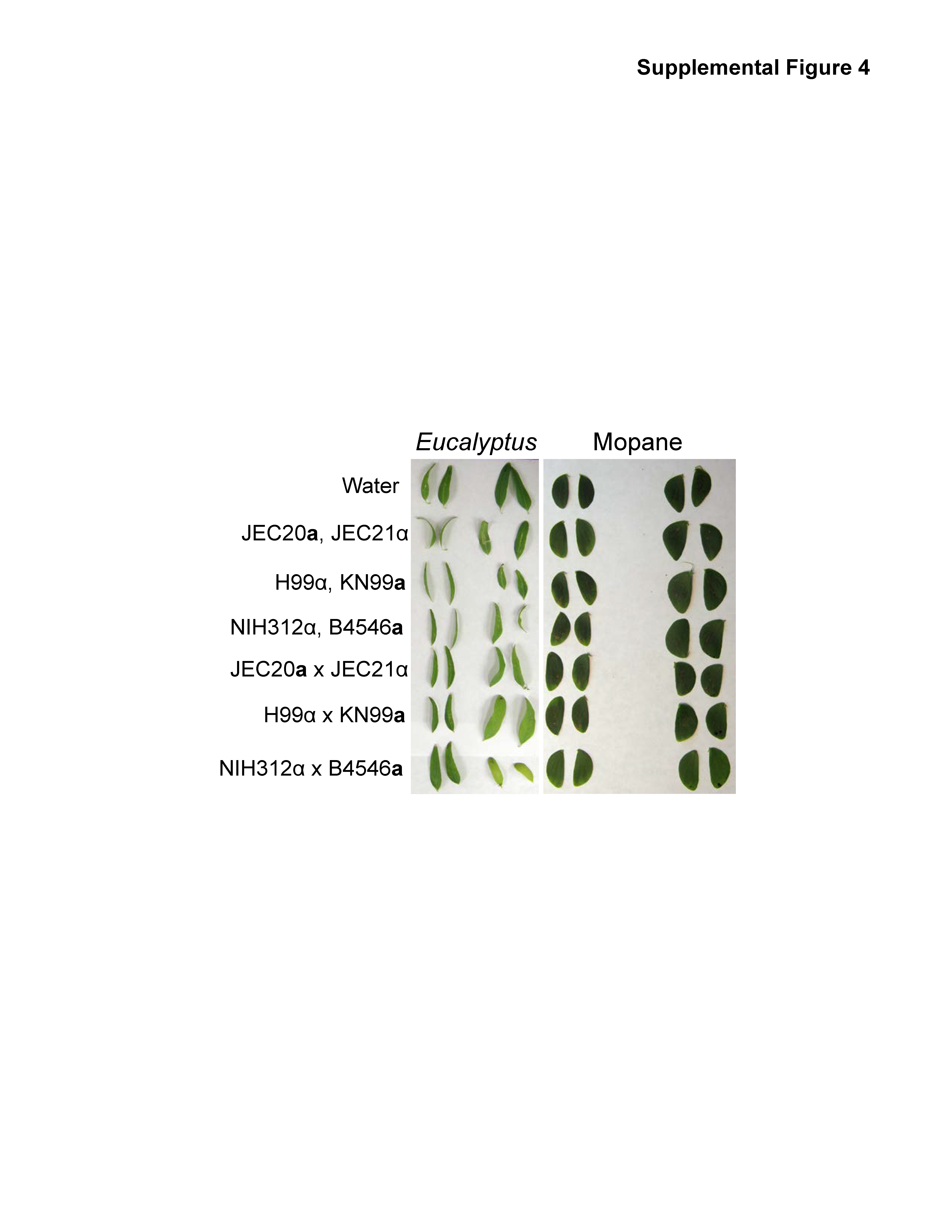

Supplement: S4 Fig — Leaves from two individual Eucalyptus or Mopane seedlings were harvested at three weeks post inoculation. (TIF) [file pone.0171695.s004.tif]

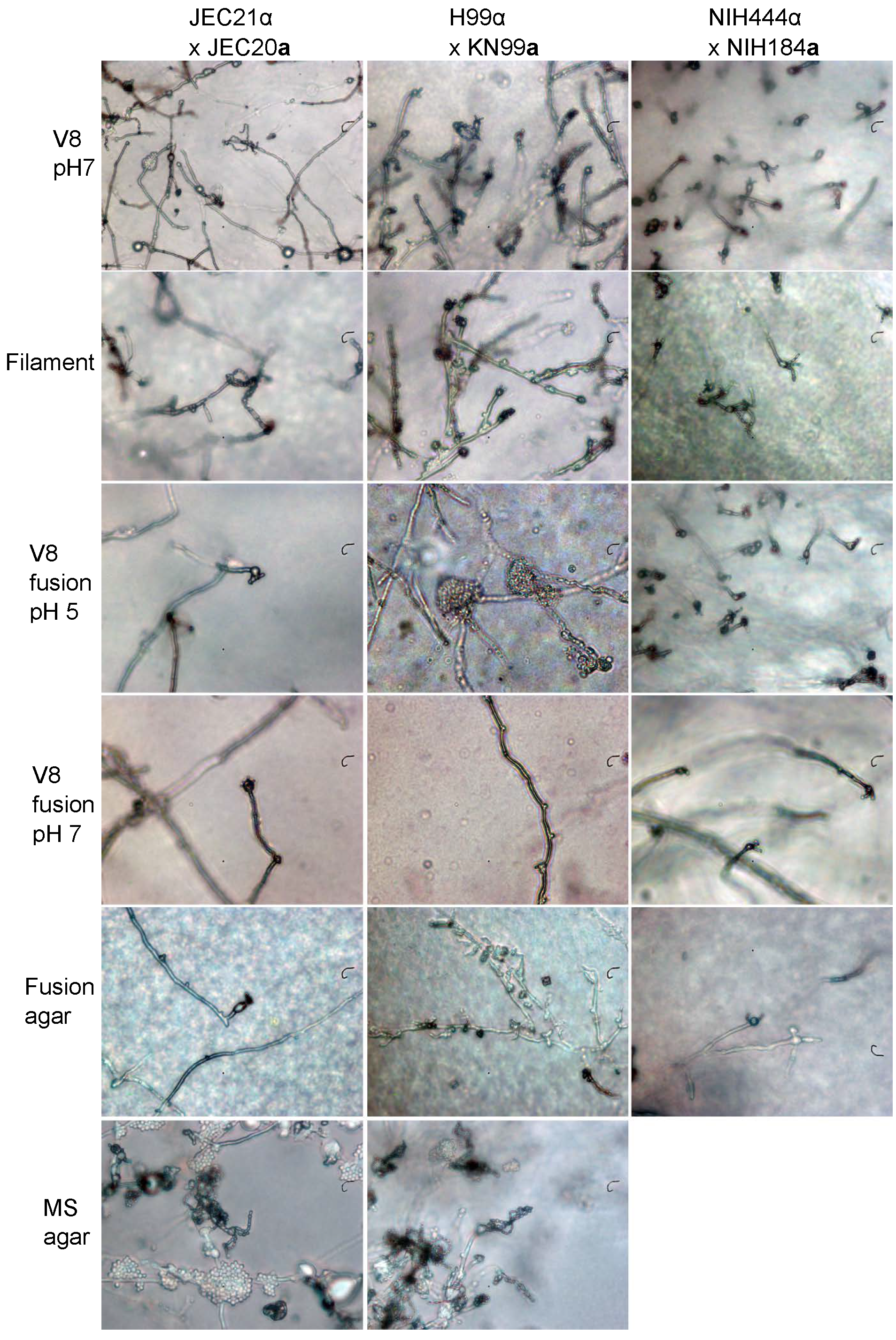

Supplement: S8 Fig — C. deuterogattii (VGII) x C. gattii (VGI), C. deneoformans, and C. neoformans robustly form basidia and spores on newly synthesized fusion and V8-fusion blend media. (TIF) [file pone.0171695.s008.tif]
