# Supplementary material for: Plants promote mating and dispersal of the human pathogenic fungus Cryptococcus
Source: PLoS One. 2017 Feb 17;12(2):e0171695. doi: 10.1371/journal.pone.0171695 (PMC5315327; doi:10.1371/journal.pone.0171695)
Supplement: S5 Fig — Robust filamentation of C. deneoformans is observed in association with black cherry chips and oak but limited filamentation is also observed in association with Sugar maple leaf, Hemlock needle, and long leaf pine needle. Robust filamentation of C. neoformans was observed in association with oak and limited filamentation with Sugar maple and Long leaf pine. (DOCX) [file pone.0171695.s005.docx]

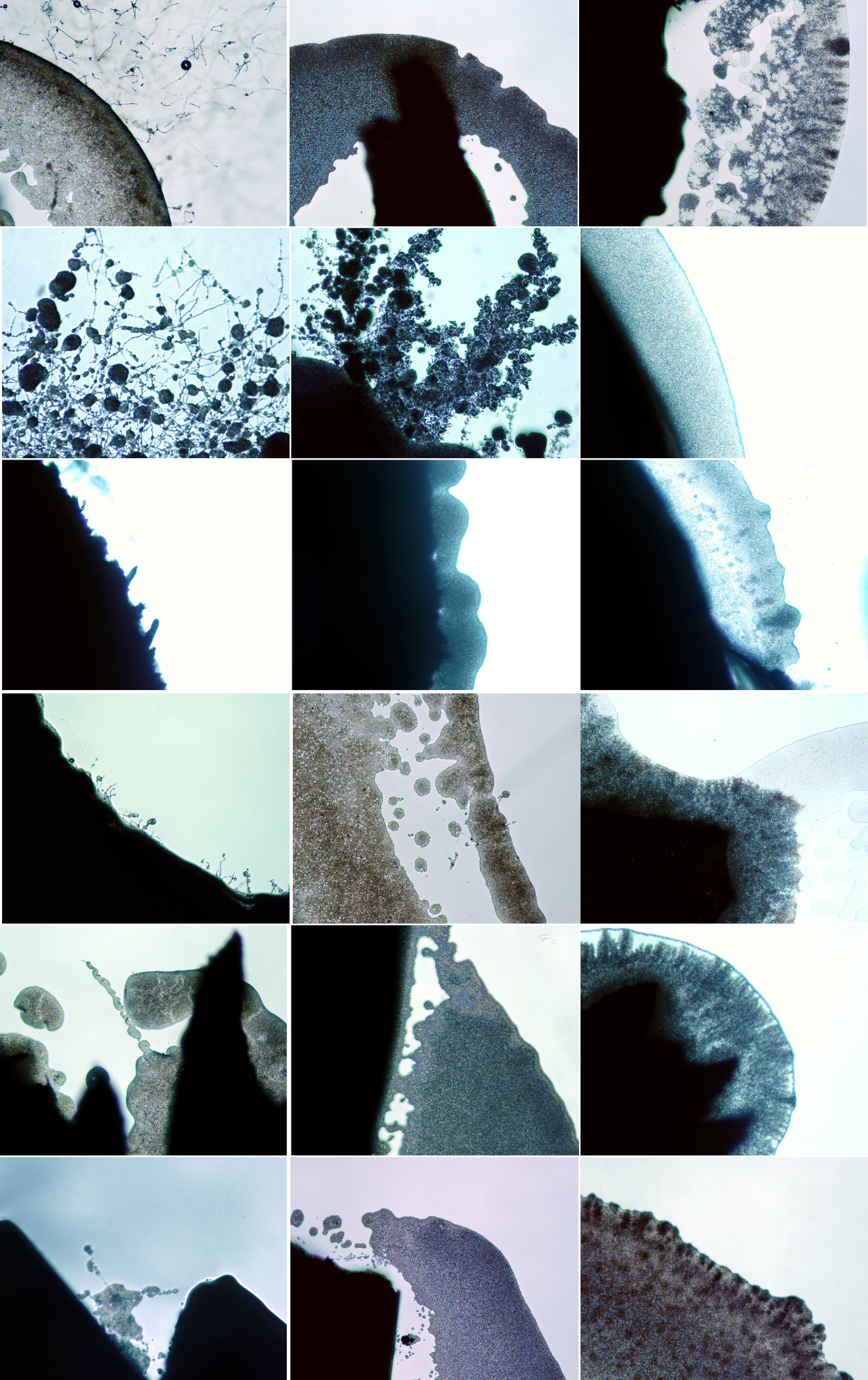


**Supplemental Figure 5**

Oak button

Pine button

Long leaf pine needle

Hemlock needle

Sugar maple leaf

Black cherry

chip

H99α

x KN99**a**

JEC21α

x JEC20**a**

NIH444α

x NIH184**a**
