# Supplementary material for: Plants promote mating and dispersal of the human pathogenic fungus Cryptococcus
Source: PLoS One. 2017 Feb 17;12(2):e0171695. doi: 10.1371/journal.pone.0171695 (PMC5315327; doi:10.1371/journal.pone.0171695)
Supplement: S6 Fig — Mating of C. neoformans is observed in as little as five to seven days post-inoculation. Detectable mating of C. bacillisporus (VGIII) x C. gattii (VGI) took longer, no filamentation or mating was observed one week post-inoculation. Red boxes denote hyphal growth and mating by light microscopic observation of basidia. (DOCX) [file pone.0171695.s006.docx]

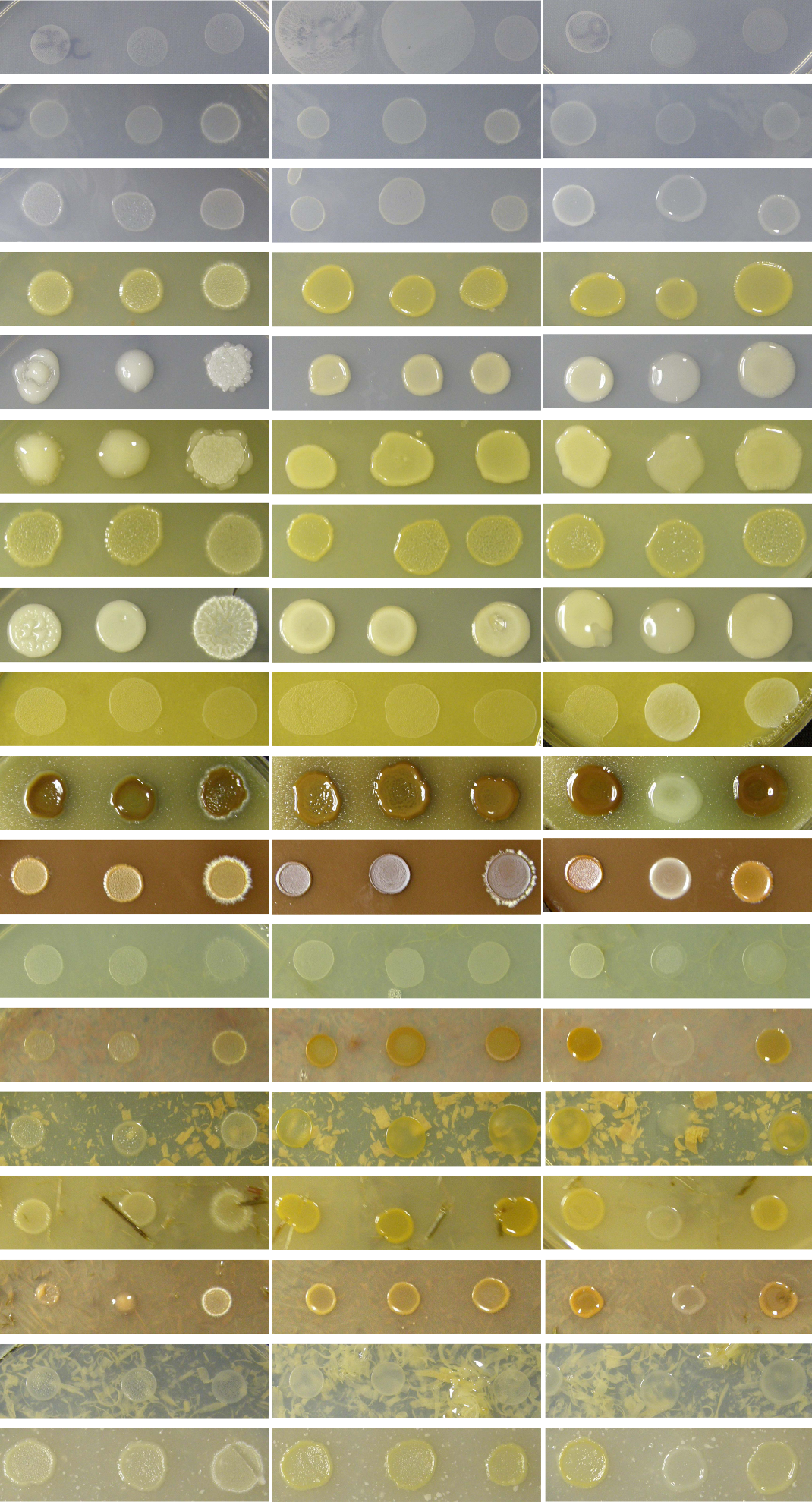


NIH312α x NIH194**a**

NIH312α

JEC21α x JEC20**a**

KN99**a**

H99α

NIH194**a**

H99α x KN99**a**

JEC20**a**

JEC21α

V8 Fusion pH 5

Almond

Hemlock

*Arabidopsis*

Coca

Black cherry

Filament

Water

MS

V8 Fusion pH 7

V8 pH 5

Fusion

Milk

Niger seed

Sugar maple

Long leaf pine

Douglas fir

V8 pH 7

**Supplemental Figure 6**
