# Supplementary material for: Plants promote mating and dispersal of the human pathogenic fungus Cryptococcus
Source: PLoS One. 2017 Feb 17;12(2):e0171695. doi: 10.1371/journal.pone.0171695 (PMC5315327; doi:10.1371/journal.pone.0171695)
Supplement: S7 Fig — C. deneoformans and C. neoformans produces filaments more prolifically than C. bacillisporus (VGIII) x C. gattii (VGI) on most media. Newly created fusion media and mixed V8-fusion media induce robust filamentation and mating of C. bacillisporus (VGIII) x C. gattii (VGI). (DOCX) [file pone.0171695.s007.docx]

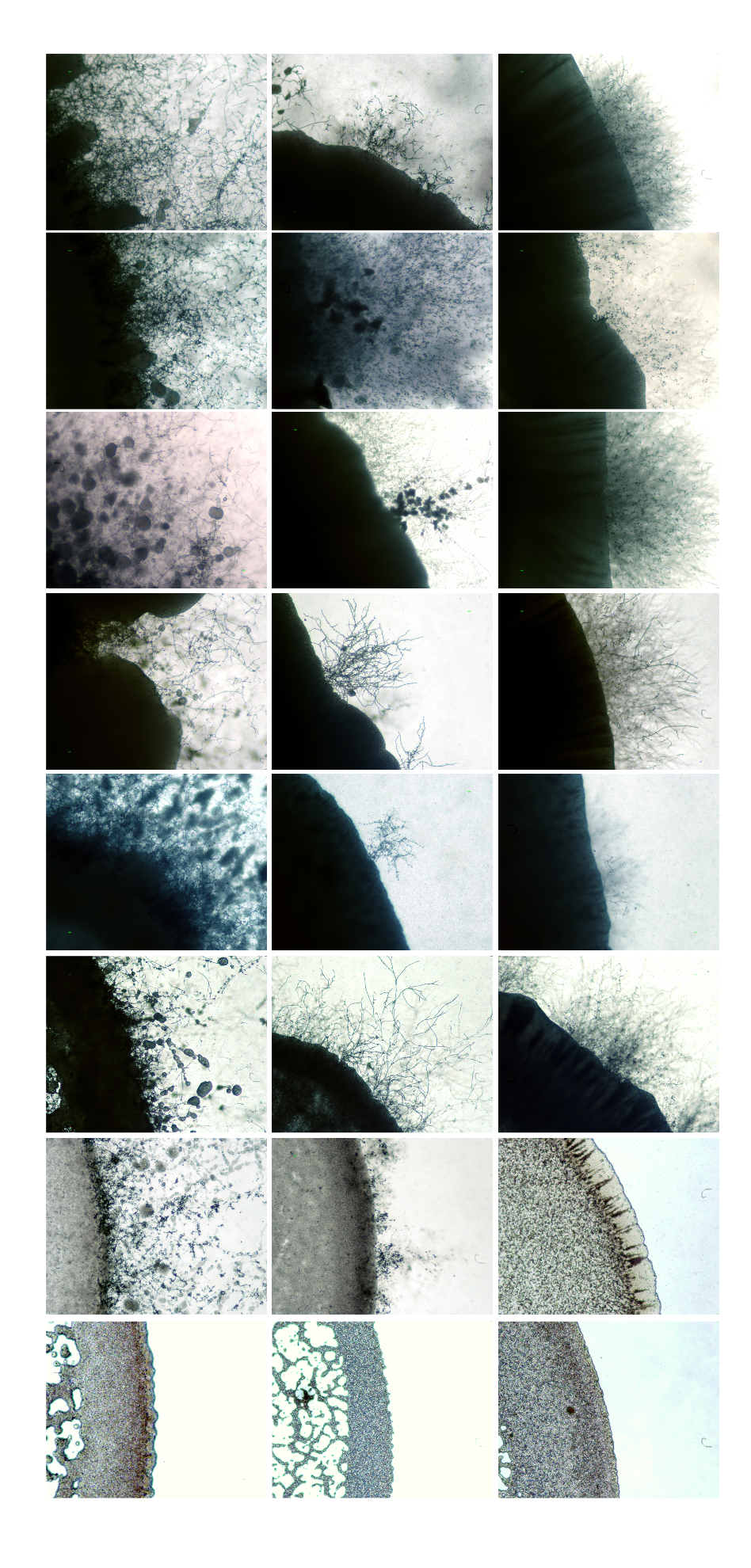


Water agar

MS

agar

Fusion

agar

V8 fusion pH5

V8 fusion

pH7

V8 pH7

Filament

agar

V8 pH5

**Supplemental Figure 7**

JEC21α

x JEC20**a**

H99α

x KN99**a**

NIH444α

x NIH184**a**
