# Supplementary material for: Plants promote mating and dispersal of the human pathogenic fungus Cryptococcus
Source: PLoS One. 2017 Feb 17;12(2):e0171695. doi: 10.1371/journal.pone.0171695 (PMC5315327; doi:10.1371/journal.pone.0171695)
Supplement: S9 Fig — C. bacillisporus (VGIII) x C. gattii (VGI) only sparsely filamented on Arabidopsis, black cherry, Coca, Long leaf pine, Sugar maple, and hemlock agars (S7 Fig). (DOCX) [file pone.0171695.s009.docx]

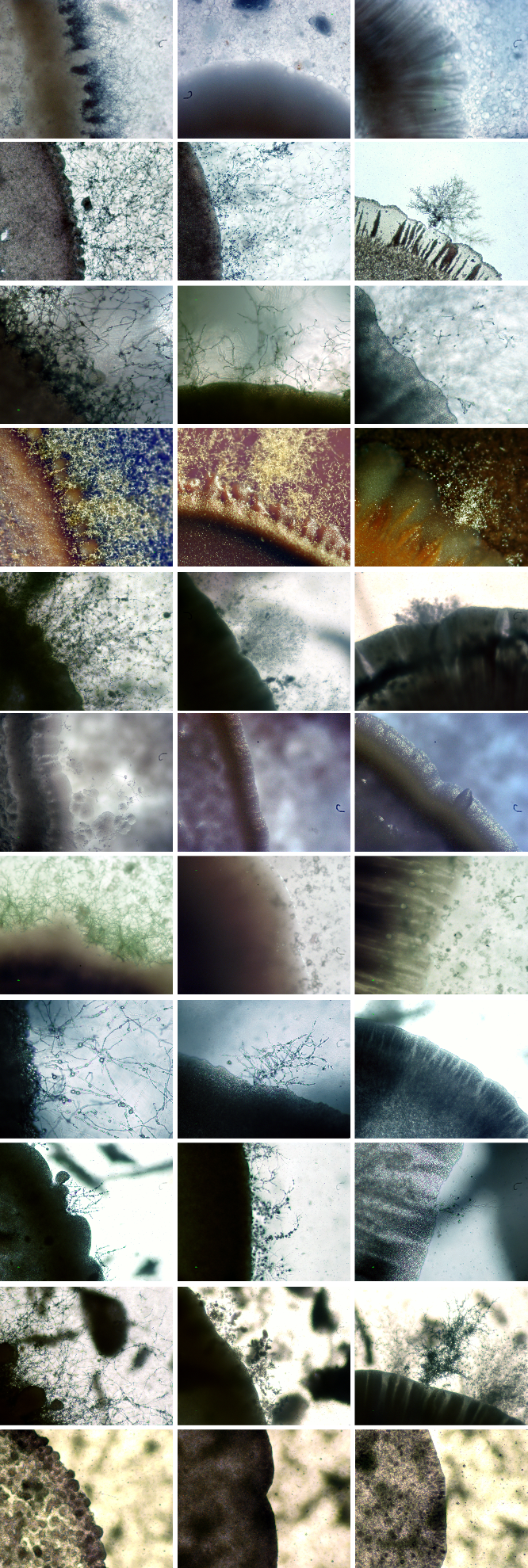


H99α

x KN99**a**

JEC21α

x JEC20**a**

NIH444α

x NIH184**a**

**Supplemental Figure 9**

Paper birch bark

Black cherry

*Arabidopsis*

Almond

Coca

Long leaf pine

Milk

Mopane

Hemlock

Sugar maple

Niger seed
