# Supplementary material for: Plants promote mating and dispersal of the human pathogenic fungus Cryptococcus
Source: PLoS One. 2017 Feb 17;12(2):e0171695. doi: 10.1371/journal.pone.0171695 (PMC5315327; doi:10.1371/journal.pone.0171695)
Supplement: S10 Fig — C. bacillisporus (VGIII) x C. gattii (VGI) also produced basidia on Arabidopsis, black cherry, Coca, Sugar maple, and hemlock agars. (DOCX) [file pone.0171695.s010.docx]

**Supplemental Figure 10**


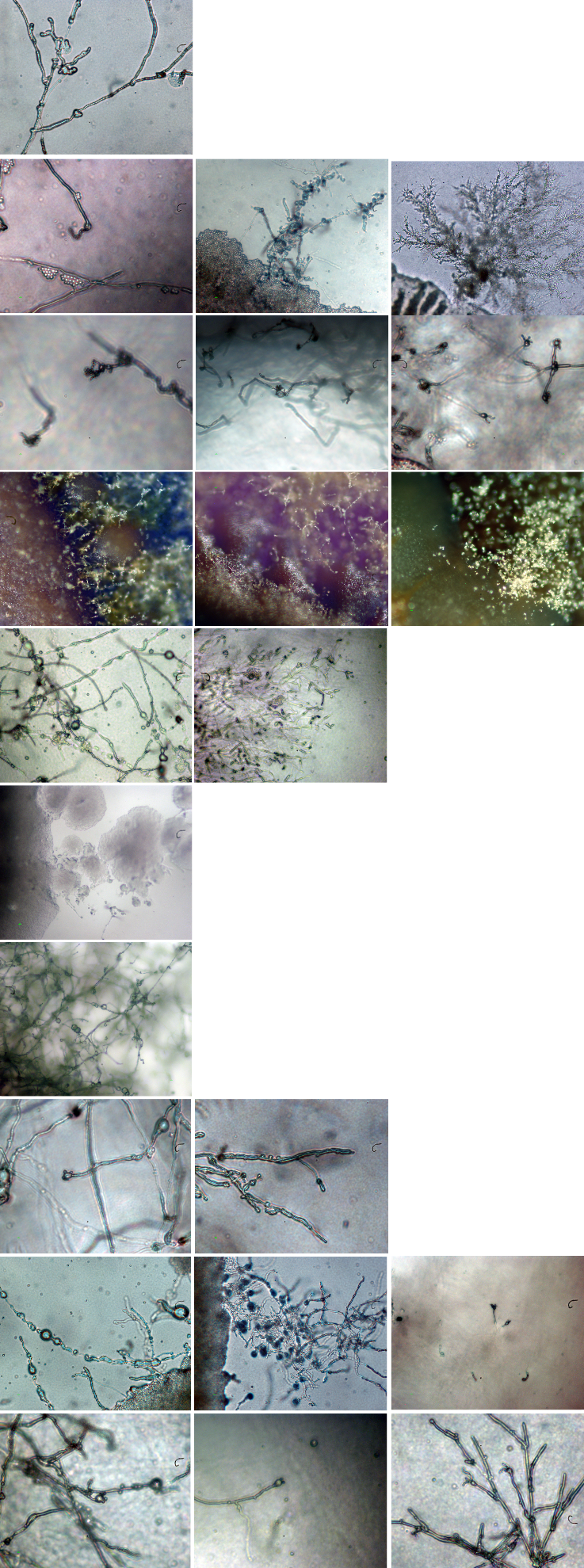


JEC21α x JEC20**a**

H99α x KN99**a**

NIH312α x NIH194**a**

Paper birch bark

Milk

Niger seed

Coco

*Arabidopsis*

Black cherry

Sugar maple

Long leaf pine

Hemlock

Almond
