# Supplementary material for: Plants promote mating and dispersal of the human pathogenic fungus Cryptococcus
Source: PLoS One. 2017 Feb 17;12(2):e0171695. doi: 10.1371/journal.pone.0171695 (PMC5315327; doi:10.1371/journal.pone.0171695)
Supplement: S11 Fig — (A) Reduced growth in Arabidopsis or Pigeon guano extract broth. (B) Colony forming units vs absorbance at OD600 confirms a higher level of viable cells in YPD broth vs Arabidopsis of pigeon guano extract broth. (DOCX) [file pone.0171695.s011.docx]

**Supplemental Figure 11**

**B**

**A**
